# Transcriptome shock invokes disruption of parental expression-conserved genes in tetraploid wheat

**DOI:** 10.1038/srep26363

**Published:** 2016-05-20

**Authors:** Huakun Zhang, Xiaowan Gou, Ai Zhang, Xutong Wang, Na Zhao, Yuzhu Dong, Linfeng Li, Bao Liu

**Affiliations:** 1 Key Laboratory of Molecular Epigenetics of the Ministry of Education (MOE), Northeast Normal University, Changchun 130024, China; 2 Department of Biology, Washington University in St. Louis, MO 63130, USA

## Abstract

Allopolyploidy often triggers phenotypic novelty and gene expression remolding in the resulting polyploids. In this study, we employed multiple phenotypic and genetic approaches to investigate the nature and consequences of allotetraploidization between A- and S-subgenome of tetraploid wheat. Results showed that karyotype of the nascent allopolyploid plants (AT2) is stable but they showed clear novelty in multiple morphological traits which might have positively contributed to the initial establishment of the tetraploids. Further microarray-based transcriptome profiling and gene-specific cDNA-pyrosequencing have documented that transcriptome shock was exceptionally strong in AT2, but a substantial proportion of the induced expression changes was rapidly stabilized in early generations. Meanwhile, both additive and nonadditive expression genes showed extensive homeolog expression remodeling and which have led to the subgenome expression dominance in leaf and young inflorescence of AT2. Through comparing the homeolog-expressing patterns between synthetic and natural tetraploid wheats, it appears that the shock-induced expression changes at both the total expression level and subgenome homeolog partitioning are evolutionarily persistent. Together, our study shed new light on how gene expression changes have rapidly occurred at the initial stage following allotetraploidization, as well as their evolutionary relevance, which may have implications for wheat improvements.

Polyploidy, or whole genome duplication (WGD), has long been considered as a driving force in the evolution and diversification of plants^1^,^2^,^3^,^4^,^5^,^6^,^7^,^8^. Polyploidy includes two major types, autopolyploidy (WGD within a single species) and allopolyploidy (WGD of two or more diverged nuclear genomes). Both types of polyploidy, but allopolyploidy in particular, are thought to provide efficient avenues for rapid generation of phenotypic novelties that may directly or indirectly contribute to the initial establishment of a nascent polyploid as well as to its evolutionary success as a new species^6^,^9^. Indeed, recent studies from diverse plant taxa have shown that allopolyploidy often causes strong and abrupt genetic and epigenetic stresses that would result in both structural and functional incompatibilities. For example, the incompatibility at gene expression level, i.e., dysregulated expression of different parental alleles, is widely observed in synthetic and natural polyploids^3^,^4^,^8^,^9^,^10^,^11^,^12^,^13^,^14^,^15^. Of these changes, gene expression nonadditivity is a common feature of polyploids and which may have played crucial roles in the adaptation and evolution of plants^14^.

The *Triticum*-*Aegilops* complex contains 13 diploid species, which belong to eight distinct but related genome groups (A, D, S, M, C, U, N and T)^16^. The A and S (also referred to as B) genomes diverged from a common ancestor some 6.5 million years ago (MYA) and then combined together via allotetraploidization about 0.5 MYA, which has led to the formation of a wild tetraploid wheat *Triticum turgidum* ssp. *dicoccoides* (BBAA) and from which the cultivated tetraploid wheat, *Triticum turgidum* ssp. *durum* (BBAA), was successfully domesticated^17^,^18^,^19^,^20^. Then, a single or a few allohexaploidization events between *Triticum turgidum* ssp. *durum* (BBAA) and goat-grass *Aegilops tauschii* (DD) has led to the establishment of the hexaploid wheat, *Triticum aestivum* (DDBBAA), which occurred less than 10,000 years ago^21^,^22^. The evolutionary history of A-, B- and D-genome makes the polyploid wheat complex an excellent system to address the effects and consequences of allopolyploidy on the evolutionary success of new species. Indeed, a series of studies based on multiple molecular approaches have demonstrated that allohexaploidization has trigged a large amount of novel genetic (i.e., gene loss) and epigenetic (i.e., alteration in cytosine methylation) changes^15^,^23^,^24^,^25^,^26^,^27^,^28^. However, the recent formation and evolution only under domestication of hexaploid wheat have largely limited its relevancy to elucidate the nature and impact of the genomic and transcriptomic shocks *per se* on the long-term evolutionary process of tetraplpid wheat. In light of this, we have recently employed RNA-seq-based transcriptome profiling to investigate the homeolog expression changes of synthetic and natural tetraploid wheats, with a specific emphasis on the fate of altered homeolog expression in the long-term evolutionary process^29^. However, due to the still incomplete reference genome in wheat, the RNA-seq-based analysis albeit has the advantages of distinguishing expression of homeologs with diagnostic SNPs, makes the analysis of global total expression changes (*i.e.*, transcriptome shock) still not as robust as microarray-based assay.

In this study, we focused on the phenotypic, karyotypic and gene expression changes at the initial stage after the allotetraploidization event between A and S genomes. Specifically, we investigated the GISH/FISH-based karyotype, basic morphologies (*i.e*., plant height, autogamy and seed dispersal) and gene expression patterns of a synthetic tetraploid (designated as AT2) (S^l^S^l^AA) with its diploid parental species *Triticum urartu* (TMU06, AA) and *Aegilops longissima* (TL05, S^l^S^l^). To further evaluate if the induced novel changes showed associations with the evolutionary success of a resulting tetraploid species, we assessed the chromosome composition, morphologies and transcriptional dynamics of a natural tetraploid species *Triticum turgidum* ssp. *dicoccoides* (TD, BBAA) and its domesticated form *Triticum turgidum* ssp. *durum* (TTR13, BBAA). We specifically investigated (1) when did the phenotypic and genetic novelties be generated and how the induced gene expression changes evolved at multiple early generations after allotetraploidization; (2) the possible relevancy of the shock-induced changes in total expression level and homeolog expression partitioning to the long-term evolutionary success of tetraploid wheats.

## Results

### Karyotype stability and phenotype novelty characterize the synthetic tetraploid wheat

**Figure 1:**
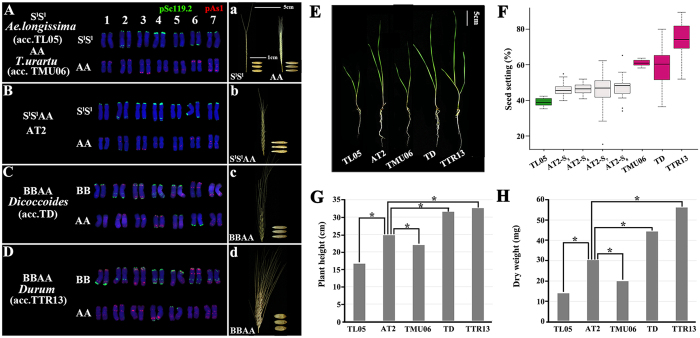
Sequential FISH-based karyotypes and typical phenotypes of the synthetic allotetraploid wheat (AT2) and its diploid parental species, along with the natural and domesticated tetraploid wheats. (**A–D**) are FISH images of the diploid parental species *Ae. longissima* (TL05) and *T*. *urartu* (TMU06), synthetic allotetraploid wheat AT2, a wild tetraploid wheat *dicoccoides* (TD), and a domesticated tetraploid wheat *durum* (TTR13), respectively. The probes used are pSc119.2 (green) and pAS1 (red). Images of (a–d) exemplify typical spike and seed morphologies of the *Aegilops* and wheat genotypes used in this study. (**E–H**) are typical seedling morphology, seed-setting rate, plant height and dry weight of AT2 relative to TL05, TMU06, TD, and TTR13. The Box blots (**F**) of different colors denote statistical significant differences (*P* < 0.05, pairwise *t* test). Statistical differences of pairwise comparisons in (**G,H**) are indicated by asterisks (*P* < 0.05, Student’s *t* test).

The study plants included the following: (1) four consecutive generations (S_5_-S_8_) of a synthetic allotetraploid wheat (AT2, S^l^S^l^AA) between diploid species *T. urartu* (TMU06, AA) –the diploid progenitor of the A-subgenome of natural tetraploid wheat *T. turgidum*, and *Ae. longissima* (TL05, S^l^S^l^)– a species of the *Sitopsis* section of *Aegilops*, presumably related to the diploid progenitor of the B-subgenome of *T. turgidum*. The choice of generations S_5_-S_8_ for AT2 was primarily based on previous studies showing that rapid genomic changes due to genome chaos in synthetic tetraploid wheats were largely stabilized after generation 5 (S_5_)^27^,^30^,^31^, and the major purpose of this study was to explore gene expression changes that are likely evolutionarily relevant; (2) A diverse collection of both natural *T. turgidum* ssp. *dicoccoides* (TD, BBAA) and domesticated *T. turgidum* ssp. *durum* (TTR13, BBAA). As revealed by the GISH/FISH analysis, karyotype of the synthetic tetraploid AT2 (S^l^S^l^AA, 2*n* = 4*x* = 28) is intrinsically stable upon formation and which is analogous to those of both subspecies, *dicoccoides* (TD) and *durum* (TTR13), of natural tetraploid wheat, *T. turgidum* (BBAA) (Fig. 1A–D). Likewise, the phenotypes of AT2 are grossly similar to the two natural tetraploid subspecies in overall plant statue, life history-related traits and spike/kernel morphology (Fig. 1a–d). For example, although the seed-setting of AT2 is significantly less productive compared to one (*T. urartu*) of its diploid parents as well as the natural tetraploid species (Fig. 1F), AT2 exhibited immediate transgressive (over-dominance) heterosis in seedling growth and biomass over both of its diploid parental species, although, expectedly, it is still inferior to the evolved natural allotetraploid species in the measured traits (Fig. 1E–H). These features make the newly formed allotetraploid AT2 a suitable system to infer the nature and consequences of changes at the initial stage after the allotetraploidization event between A and B genomes leading to formation of natural tetraploid wheat, *T. turgidum*.

### Transcriptome shock disrupts parental gene expression in the synthetic tetraploid wheat but the remodeled expression patterns showed rapid transgenerational stabilization

**Figure 2:**
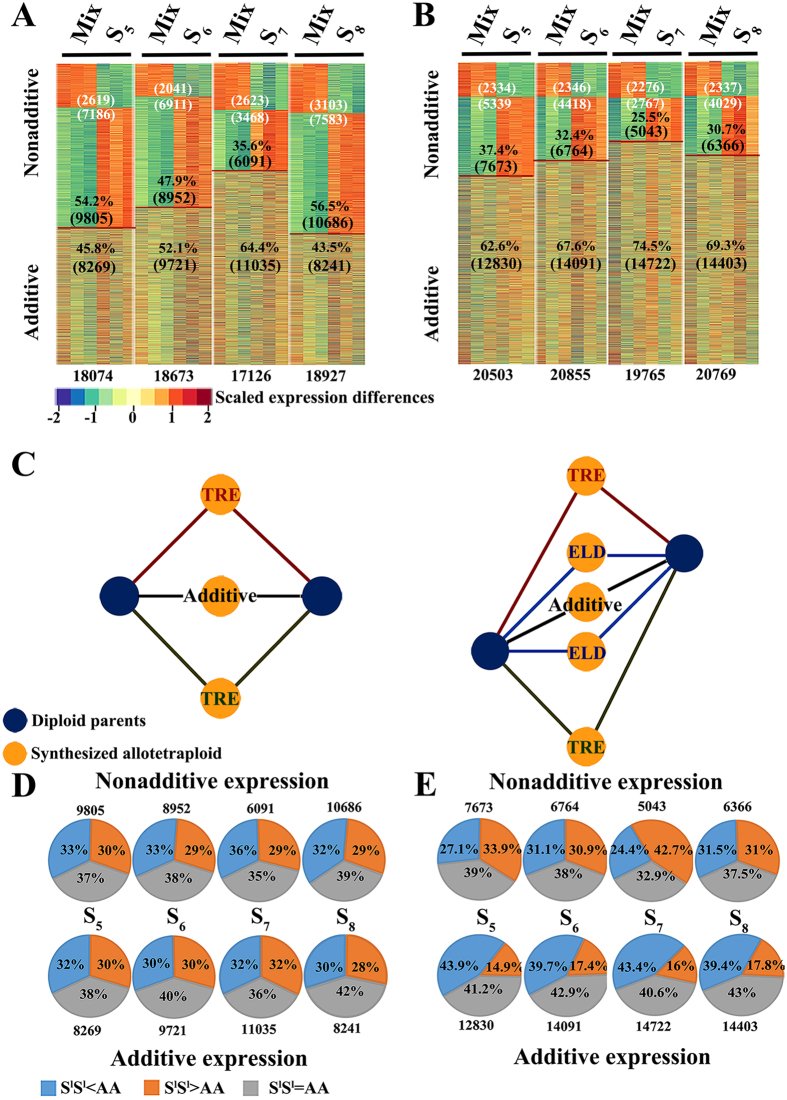
Summary of microarray-based nonadditive and additive gene expression in AT2 including the four major expression patterns and their relationships with parental expression conditions. Nonadditive and additive expressing genes in leaf (**A**) and young-inflorescence (**B**) of AT2 at generations S_5_ to S_8_ are shown. Numbers and proportions of the nonadditive (above the red lines) and additive (below the red lines) expressing genes are indicated. Numbers of the total expressing genes are shown at the bottom. Different expression space by the parental expression-conserved genes (PEC) and the parental expression-different genes (PED) in AT2 are diagrammed (**C**). PEC genes can exhibit additive expression and transgressive expression (TRE), while, apart from additive expression and TRE, PED genes may also show higher or lower parental expression level dominance (ELD). The proportions of the non-additive and additive genes that are PEC (S^l^S^l^ = AA) or PED (S^l^S^l^ ≠ AA) in leaf (**D**) and young-inflorescence (**E**) of AT2 from S_5_ to S_8_ are shown by pie charts. Each pie represents the total number of the nonadditive or additive genes at a given generation of AT2, which was divided into three colored parts based on its relationships with parental expression conditions.

**Figure 3:**
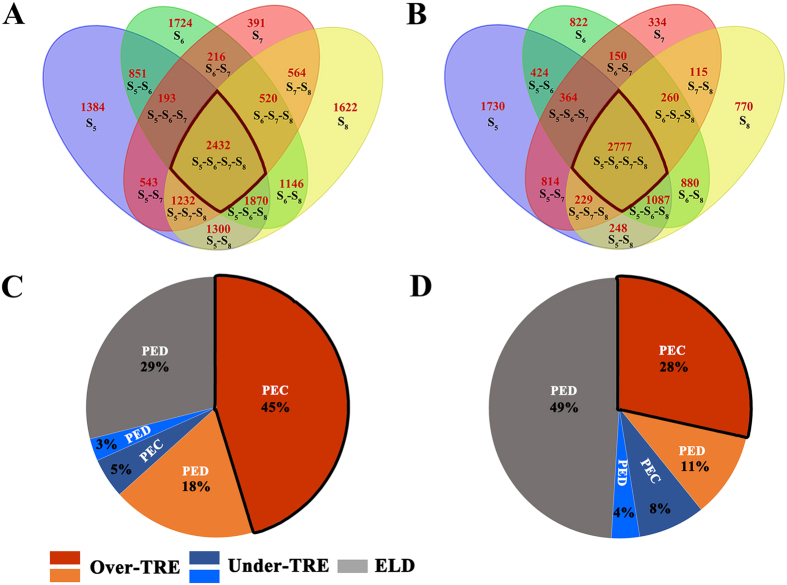
Numbers and proportions of the transgenerational conserved nonadditive expressed genes, and sources of those genes in AT2. (**A**,**B**) are the numbers of nonadditive expressing genes that are transgenerational conserved nonadditive expressed or no-transgenerational conserved nonadditive expressed genes in leaf and young-inflorescence of AT2, respectively. The four color ellipses represent the four early generations of AT2. There are two sources for transgenerational conserved nonadditive expressed genes, i.e., parental expression conserved genes (PEC) and parental expression different genes (PED). The PEC genes can exhibit only two patterns of nonadditive expression, i.e., over- or under-transgressive expression or TRE, while four patterns of nonadditive expression are possible for the PED genes, that is, apart from the two patterns of TRE, PED genes may also show higher or lower parental expression level dominance or ELD. The transgenerational conserved nonadditive expressed genes of both leaf (**A**) and young-inflorescence (**B**) can be further divided into several subclasses, as illustrated by pie charts (**C**,**D**), respectively.

Based on the Affymetrix GeneChip® Wheat Genome Array (The Affymetrix, Inc., Santa Clara, CA, USA) profiling, we detected 16,275 and 19,204 genes that showed reliable expression (based on MAS5 flags analysis for reproducibility among the three biological replications for a given genotype) in the leaf and young-inflorescence tissues, respectively. Through comparing the whole transcriptome expression difference between the two diploid parental species, we found that a total of 73% and 67% genes are differently expressed, which were defined as parental expression-differential (PED) genes in the leaf and young inflorescence, respectively (Fig. S1). The rest genes showed equal expression between the parental species in the respective tissues, and which were defined as parental expression-conserved (PEC) genes. An interesting observation is that the proportions of PED and PEC genes are statistically different between the two tissues (prop.est *P* < 2.2e-16) (Fig. S1). In addition, we also found that, of the PED genes, the proportion of up-regulated genes were significantly greater (prop.test, *P* < 0.05) than that of the down-regulated genes in young inflorescence, but both of up- and down-regulated genes are statistically equal in leaf (prop.test, *P* > 0.05) (Fig. S1). Together, the results suggest that with regard to total gene expression level, both PEC and PED genes between the two diploid parental species showed tissue-specific differences. We then compared the relative expression level of each gene with the mid-parent values (MPVs) and found that substantial proportions of genes showed nonadditive expression in both tissues at each of the four consecutive early selfed generations (S_5_ to S_8_) of the synthetic allotetraploid wheat AT2, indicating that a strong transcriptome shock was invoked upon the formation of AT2 (Fig. 2A,B). Nevertheless, in contrast to widely accepted conclusions from previous studies and despite the unequal spaces for the PEC and PED genes to show nonadditive expression in the resulting allopolyploids (Fig. 2C), we found that these two gene categories, PEC and PED, showed statistically equal propensities for nonadditive *vs* additive expression (prop.test, *P* > 0.05) in both tissues at each of the four generations of AT2 (Fig. 2D,E). This observation suggests that although strong transcriptome shock was invoked in the nascent allotetraploid (AT2), some of the rapid expression changes have already been stabilized and maintained across the four generations. We therefore evaluated how many genes have shown the same nonadditive expression patterns across the early generations following the transcriptome shock, and if the shock *per se* is still transgenerationally ongoing and affecting different genes in different generations of AT2. We found that a total of 2,432 (in leaf) and 2,777 (in young inflorescence) genes showed the same nonadditive expression pattern across the four consecutive generations in AT2 (Fig. 3A,B). These transgenerationally consistent nonadditive expressing genes accounted for 23–40% (in leaf) and 36–55% (in young inflorescence) of the total genes showing nonadditive expression in each of the four generations (Fig. 3A,B). Notably, the proportions of nonadditive expressing genes in young inflorescence were significantly higher than those in leaf (prop.test, *P* < 0.05), suggesting difference between the two tissues in rapid stabilization of the shock-induced gene expression rewiring.

### Diverse expression patterns contribute to the additive and nonadditive expression genes in the synthetic tetraploid wheat

In theory, various patterns of gene expression may contribute to each of the additive and nonadditive expression categories. Specifically, the PEC genes can exhibit only two patterns of nonadditive expression, *i.e*., over- or under-transgressive expression or TRE (i.e., expression levels in the allopolyploid are significantly higher or lower than those of both parents), while four patterns of nonadditive expression are possible for the PED genes (Fig. 2C). We thus analyzed the additive and nonadditive expression patterns at each of the four generations (S_5_ to S_8_) in both tissues of AT2 (Fig. S2). We found that the two categories of additive expression genes, constant expression of PEC and additive expression of PED, constitute 43.5–64.4% and 62.6–74.5% of the total genes in leaf and young inflorescence, respectively (Fig. 2A,B). Of the nonadditive expression genes, we made the following observations: (*i*) two major categories, ELD (parental expression level dominance) and TRE genes, are comprised of the nonadditive expression genes in AT2; (*ii*) smaller numbers of ELD genes than TRE genes were observed in both tissues; (*iii*) TRE was contributed more by the PEC than by the PED genes; (*iv*) more over- than under-TRE genes were detected; (*v*) of the ELD genes, similar proportions were contributed by either diploid parent.

Given that the transgenerationally consistent nonadditively expressed genes are comprised of two major sources, ELD and TRE, we assessed the relative proportions of these two sources in each tissue of AT2 (Fig. 3C,D). We found that the relative proportions of ELD and TRE genes contributing to the transgenerationally consistent nonadditively expressed genes were variable in the two tissues: while in leaf, TRE contributed more than ELD (Fig. 3C, prop. test, *P* < 0.05), in young-inflorescence, TRE and ELD contributed equally (Fig. 3D, prop. test, *P* > 0.05). In both tissues, however, majority of the transgenerationally consistent TRE genes were over-TRE, and most of them were sourced from the PEC genes rather than the PED genes (Fig. 3C,D). We conducted a gene ontology (GO) analysis for this specific category of the transgenerationally consistent nonadditively expressed genes, *i.e.*, the PEC-derived over-TRE genes of each tissue (Table S1). We found that these genes were highly enriched for protein transport/modification and uroporphyrinogen decarboxylase activity, in leaf and young inflorescence, respectively. This suggests that the genes showing transgenerational over-transgressive expression at early generations of allopolyploidy likely bears very specific biological functions in a given tissue.

### Transcriptome shock induces extensive remodeling of homeolog expression in the synthetic tetraploid wheat

**Figure 4:**
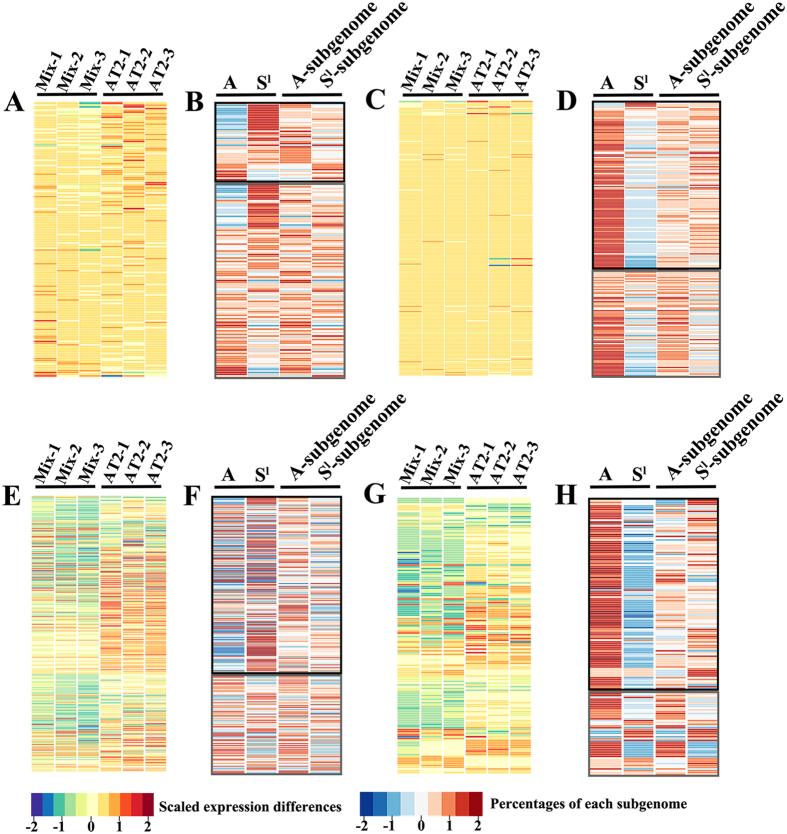
Clustering of microarray data-based nonadditive and additive expressing genes in AT2 relative to its MPVs (*see* in Method and Materials) as total expression (all three biological replicates are shown) in relation to the cDNA pyrosequencing-based relative transcript contribution by the A and S^l^ subgenomes for each studied gene in leaf and young-inflorescence, respectively. The upper panels are the results of additive expression genes in leaf (**A,B**) and young-inflorescence (**C,D**), respectively, while the lower panels are the results of nonadditive expression genes in leaf (**E,F**) and young-inflorescence (**G,H**), respectively. Altered partitioning by the A vs. S^l^ subgenome transcripts in AT2relative to the original parental ortholog transcript proportion in the *in vitro* “hybrids” were determined by statistical analysis: *t* test, *P* < 0.05 for altered partitioning (framed by black rectangles), and *P* > 0.05 for unaltered partitioning (framed by grey rectangles). In total, 94 and 95 additive expression genes were analyzed for in leaf and young-inflorescence, respectively, while 125 and 67 nonadditive expression genes were analyzed for leaf and young-inflorescence, respectively. The color key is indicated.

The foregoing results have documented the occurrence of transcriptome shock at the total gene expression level in the synthetic tetraploid wheat AT2. To further interrogate the homeolog expression status (*i.e*., altered homeolog expression partitioning) of the A- and B-subgenome for a given gene, the locus-specific cDNA-pyrosequencing was applied to analyze the relative homeolog expression changes (compared to the relative expression by parental orthologs in the *in vitro* “hybrids”) for a subset of randomly chosen unique-copy genes for which the analysis could be applied. To this end, a total of 94 additive (showing additive expression in at least one of the four studied generations) and 125 nonadditive (showing nonadditive expression in at least one of the four studied generations) expressing genes in leaf and 95 additive and 67 nonadditive expressed genes in young inflorescence were investigated, respectively. For the additive expressing genes in leaf and young-inflorescence, the corresponding numbers of the comparable data points (*see* Method and Material) were 159 and 208. Apparently, the numbers of the actual comparable data points are less than theoretically possible; this is because a given gene may not show additive expression in all the four studied generations (S_5_-S_8_). We found that 28.9% (46/159) and 60% (126/208) of the comparable data points showed altered expression ratios in leaf and young inflorescence, respectively (Fig. 4A–D; Data Set S1). These two proportions of altered homeolog expression partitioning for the additive expressing genes in the two tissues differed significantly (prop. test, *P* < 0.05). For the nonadditive expressing genes, of the 125 genes analyzed in leaf of AT2, homeologs in 188 of the 291 (64.6%) comparable data points showed altered expression ratios, while of the 67 genes analyzed in young inflorescence of AT2, 100 of the 154 (64.9%) comparable data points showed altered expression ratios (Fig. 4E–H; Data Set S1). These two proportions of altered homeolog expression partitioning for the nonadditive expression genes in the two tissues are statistically equal (prop. test, *P* > 0.05).

### The rapidly established overall subgenome expression dominance in the synthetic tetraploid wheat was mirrored and augmented by natural tetraploid wheats

There are two emergent qualitative outcomes of remodeled homeolog expression partitioning, namely, erasure of original biased expression of the diploid parental orthologs and establishment of novel biased homeolog expression in the synthetic allotetraploid (AT2). To assess the magnitude of these two contrasting qualitative outcomes, we analyzed 150 genes by cDNA-pyrosequencing in each of the four early generations (S_5_ to S_8_) of AT2 for both tissues. We found that in leaf of AT2, from 35% to 56% (depending on generation) of the analyzed genes showed stable inheritance of the parental ortholog expression ratios seen in the *in vitro* “hybrids”, while the rest 44–65% genes showed remodeled homeolog expression partitioning (Table S2). Of these homeolog-expression remodeled genes in leaf, 24–43% were erasure of original biased expression of parental orthologs (hence, resulting in equal homeolog expression in AT2), while the rest 11.5–28.3% were novel biased homeolog expression in AT2 (Table S2), the two types of homeolog expression alterations produced the net consequence of reduced homeolog expression bias in AT2. Extensive remodeling of homeolog expression partitioning was also observed in young inflorescence, in which 43–49% of the analyzed genes showed stable inheritance of the parental ortholog expression ratios seen in the *in vitro* “hybrids”, while the rest 51–57% genes showed alteration of homeolog expression partitioning (Table S3). Of the remodeled genes in this tissue, 29.2–39.4% were erasure of original biased expression of parental orthologs (hence, resulting in equal homeolog expression in AT2), while the rest 21–31% were novel biased homeolog expression in AT2 (Table S3), the two types of homeolog expression alterations also produced the net consequence of reduced homeolog expression bias in AT2. Together, these results indicate that the remodeled homeolog expression partitioning largely occurred in the analyzed genes in both tissues of the synthetic allotetraploid wheat (AT2) at each of the four (S_5_ to S_8_) early generations following allotetraploidization. In both tissues, the resulting extent of homeolog expression bias is smaller than the original parental ortholog expression bias in the *in vitro* “hybrids” (Tables S2 and S3). Notwithstanding this overall pacifying effect of homeolog expression partitioning, it resulted in an overall A-subgenome biased expression at the homeolog expression level in both tissues of AT2 when all the analyzed genes were considered together (Tables S2 and S3).

**Figure 5:**
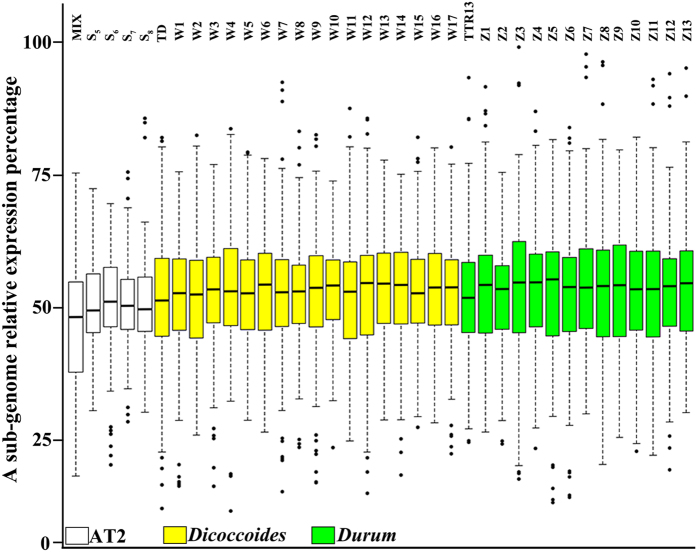
Subgenome expression divergence in AT2 and a set of wild (*dicoccoides*) and domesticated (*durum*) tetraploid wheat genotypes in leaf. cDNA pyrosequencing-based gene expression spectra of subgenome homeolog expression divergence for a total of 66 genes in the *in vitro* “hybrids”, the S_5_-S_8_ generations of the synthetic allotetraploid wheat AT2, 18 accessions of *dicoccoides* and 14 cultivars of *durum*, revealed by a boxplot. The color key is indicated.

To test if the transcriptome shock-induced emergent overall biased subgenome expression in the synthetic tetraploid wheat might bear evolutionary relevancy, we examined the homeolog expression partitioning for a subset of the genes (66 in total) by cDNA-pyrosequencing in the leaf tissue of a set of 18 wild (*dicoccoides*) and 14 domesticated (*durum*) genotypes of the nature tetraploid wheat, *T. turgidum*, of diverse origins (Table S4). We found that although for this subset of genes (66 in total), overall expression bias by one subgenome in leaf was not consistently manifested by all four generations of AT2, the trend towards higher A- than S^l^-subgenome homeolog expression is clear in comparison with the *in vitro* “hybrids” (mix) (Fig. 5). Moreover, as described above, when all the analyzed genes were considered together, the trend is even more evident (Table S2). Strikingly, all analyzed natural tetraploid wheat genotypes showed the overall A subgenome homeolog-biased expression (Fig. 5) at extents significantly greater than that in the synthetic (Table S2). This suggests that the transcriptome shock-induced remodeling of homeolog expression partitioning culminating to subgenome expression dominance has evolutionary consequences and likely being selected for and further augmented by both natural and human selections.

## Discussion

### Phenotypic and gene expression novelties in the synthetic tetraploid wheat

It is widely accepted that allopolyploidization is a driving evolutionary force known to generate instantaneous and sometimes even saltational genome-scale genetic and epigenetic changes^13^. Laboratory synthesized allopolyploids with exact parentage while mimicking natural species in genome composition represent a suitable system to study the phenomenon^32^. In this study, we have employed phenotypic, karyotypic and molecular genetic approaches to evaluate the phenotypic and gene expression novelties in two natural (TD and TTR13) and one synthetic (AT2) tetraploid wheats.

It has been observed in diverse plants that the immediate consequence of allopolyploidization often cause fitness reduction with respect to the fertility^13^. For example, about 20% reduction in pollen viability and 50% reduction in seed production were observed in some newly formed plant polyploids^33^. On the other hand, polyploids also potentially benefit from “hybrid vigor” which make them immediately superior to both parental species and eventually lead to successful speciation^13^. For example, it was found that in wild yarrow, about 70% fitness advantage can be achieved via genome duplication *per se*^34^. Here, our phenotypic analyses revealed that the newly formed tetraploid wheat AT2 shows an “additive” pattern of the reproductive fitness (*i.e*., seed setting) at the early generations compared to the two parental species, but both of the diploid and synthetic tetraploid wheats show significantly lower reproductive fitness than those of the two natural tetraploid species. These observations confirmed previous findings in that the early generation polyploids have negative effects on fertility, but our data suggest that this could be improved progressively in the long-term evolutionary process^33^,^35^,^36^. Moreover, we showed that the early generations of AT2 exhibit immediate heterosis in seedling growth and biomass over its diploid parents, although both traits in AT2 are significantly lower than those of the two natural tetraploid subspecies. These features suggest that “hybrid vigor” could be generated immediately after allotetraploidization in wheat and which might have positively contributed to the initial establishment of the nascent tetraploid species as a competitive population.

The observed phenotypic novelties in the synthetic tetraploid wheat AT2 suggest that rapid changes in gene expression, *i.e.*, transcriptome shock, is likely a major cause. This is because karyotype of AT2 is intrinsically stable, therefore aneuploidy and large-scale chromosome structural changes can be ruled out as a main cause for its phenotypic changes. Notably, we found that the transcriptome shock observed in AT2 is much stronger than those reported in the hexaploid wheat^15^,^23^,^24^,^25^,^26^,^28^,^37^. For example, the proportion of genes showing nonadditive expression in AT2 can be as high as 56.5% in leaf of generation S_8_. Most unexpectedly, we found that a major cause for the significantly higher proportion of nonadditively expressed genes in AT2 is due to a large number of PEC genes being disrupted and remodeled to show nonadditive expression. This observation also contrasted sharply with the situations of hexaploid wheat in which the source of nonadditive expressing genes was mainly from the PED genes; so are studies in other plant taxa. For instance, about 70% of the nonadditive expression genes are from the PED genes in two independent synthetic hexaploid wheat lines^26^, while the proportions in *Arabidopsis*^38^ and cotton^39^ are ~68% and ~85%, respectively. Thus, our findings suggest that the formation of allotetraploid wheat is associated with an exceptionally strong transcriptome shock, which is unprecedented in hexaploid wheat and other plant allopolyploids studied.

We have reported recently that there are two distinct stages for the evolutionary changes of homeolog expression associated with tetraploid wheat evolution^29^. However, for the synthetic tetraploid wheat, plants of only one generation were investigated in the study^29^. Here, by comparing gene expression patterns of four consecutive early generations of AT2, we show that although extensive transcriptome shock has occurred in AT2, these gene expression changes can be rapidly stabilized and transgenerationally heritable. In particular, the majority of these genes belong to the over-TRE (transgressive expression) category, which have been documented in both our GO analyses and previous studies as often associated with heterotic growth and biomass of F1 hybrids and allopolyploids^8^,^40^,^41^,^42^,^43^. Taken together, these observations allow us to propose that the transcriptome shock of allotetraploidization of this specific combination, albeit exceptionally strong, may have positively contributed to the initial establishment of the newly formed plants as a competitive population, and eventually contributes to speciation of tetraploid wheats.

### Homeolog expression remodeling and subgenome expression dominance

Homeolog expression remodeling and subgenome expression asymmetry are common features of plant allopolyploids and which are thought to play important roles in the evolutionary success of the allopolyploid species^9^,^14^,^28^,^44^,^45^,^46^,^47^,^48^,^49^. Using RNA-seq-based transcriptome-profiling, we have revealed in our previous study that homeolog expression rewiring shows a tissue-specific manner in tetraploid wheats^29^. Here, the microarray-based transcriptome analysis and cDNA-pyrosequencing on two tissues (leaf and young-inflorescence) of four consecutive generations of AT2 further showed that extensive remodeling in homeolog expression partitioning occurred in each tissue of AT2 for both additive and nonadditive expressing genes. While homeolog expression partitioning is expected for the nonadditive expression genes, the phenomenon is unexpected for the additive expression genes, because to maintain the additive expression pattern at total expression level would entail coordinated expression changes of homeologs. One possible explanation is that the exceptionally strong transcriptome shock might have led to re-programming of homeolog expression pattern in both additive and nonadditive expressing genes. Theoretically, the PEC genes are likely functionally essential, which are often under the same *cis*-regulation between the two parental species due to preferential fixation of *cis*-regulatory mutations than those of *trans*^50^. Thus, the PEC genes might be more likely to show additive expression in the resulting allotetraploids^38^,^51^,^52^. Indeed, it was suggested that genes controlled by *cis*-acting factors alone (*cis*-only) tended to show additive expression in allopolyploids^14^. However, our results revealed that a majority of these PEC genes were expressed as TRE in the newly formed tetraploid, possibly due to the compensatory interactions of *cis*-/*trans*-regulators and/or gene dosage constraint^44^,^53^. In addition, we noted that, of the additive expression genes, the extents of altered homeolog partitioning showed between-tissue difference, which was much greater in young inflorescence than in leaf. However, there was no difference in the extents of remodeled homeolog expression partitioning for the nonadditive expression genes between leaf and young inflorescence. These findings implicate another layer of intricacy that can be explored by allopolyploidy to generate tissue-specific novel gene expression patterns.

We have shown previously that a re-established subgenome expression asymmetry has occurred in the young inflorescence of natural tetraploid wheats^29^. To this end, it is of apparent interest to explore whether this subgenome asymmetry was already established at the initial stages of allotetraploidization or a long-term evolutionary outcome. Our results here document that, in the leaf tissue, subgenome A showed immediate expression dominance over subgenome S^l^ (~B) following the transcriptome shock. In particular, we show that this shock-induced biased subgenome expression not only mirrored that seen in natural tetraploid species but also showed evidence of reinforcement in all studied wild and cultivated natural tetraploid wheat genotypes of diverse origins. These findings are consistent with our previous observations that, in leaf, transcriptome shock likely has played an instantaneous as well as protracted role in determining the subgenome expression status (dominant *vs* subordinate) both at the initial stage after allotetraploidization and in the course of allopolyploid genome evolution^29^. It should be noted however that the cDNA-pyrosequencing of the selected genes revealed A-subgenome dominance in the young inflorescence of AT2, which appeared as not in full accordance with results of the RNA-seq-based transcriptome profiling^29^. This appeared discrepancy can be partially explained by the limited number of loci that can be assayed by pyrosequencing in tetraploid wheat^54^. Also, we noted that as much as 74.7% of the analyzed DNA-pyrosequencing loci showed A-bias in the *in vitro* “hybrids”, indicating majority of this set of genes are intrinsically expressed at higher levels in the A-genome diploid parent, *T. urartu*, than in the S-genome parent, *Ae. longissima*. Thus, the proportion of A-biased genes is obviously reduced in the four early generations of AT2, due to increased numbers of B- biased and no-biased genes. These findings, coupled with our previous RNA-seq-based transcriptome-profiling^29^, suggest that although evolution and domestication under allotetraploidy have led to the re-establishment of A-subgenome expression asymmetry in the natural tetraploid wheats, the B-subgenome expression asymmetry has been generated immediately after allotetraploidization followed by persistent maintenance. Given that the natural and artificial selections might have favored higher A-subgenome expression genes in the tetraploid wheats during the evolution and domestication processes^28^,^29^,^45^,^47^, this issue warrants further investigations because understanding the time point of the re-establishment of subgenome asymmetry may provide important clues for more judicious utilization of synthetic wheats for breeding purposes^55^.

## Methods

### Plant Material

Seeds at selfed generation 2 (S_2_) of the synthetic allotetraploid wheat (designated AT2) and their parental species, *Ae. longissima* (genome S^l^S^l^, accession TL05) and *T. urartu* (genome AA, accession TMU06) were procured from Dr. Moshe Feldman (Weizmann Institute of Science, Israel). AT2 (2*n* = 4× = 28, genome S^l^S^l^AA) was produced by intergeneric hybridization followed by colchicine-mediated genome doubling of the first filial generation (F1) hybrids between TL05 (♀) and TMU06 (♂). AT2 and its parental genotypes were propagated under strict selfing to obtain seeds of advanced generations. The collected seeds of 18 natural wild tetraploid wheat, *T. turgidum ssp.* *dicoccoides* (2*n* = 4× = 28, genome BBAA) and 14 domesticated form of *T. turgidum ssp. durum* (2*n* = 4× = 28, genome BBAA) were also propagated by strict selfing. Detailed information on these plants was provided (Table S4).

### Sequential FISH and GISH

The protocols for florescent *in situ* hybridization (FISH) and genomic *in situ* hybridization (GISH) were essentially as described^56^ with minor modifications^54^.

### Microarray Hybridization

Total RNA was extracted using Trizol reagent (Invitrogen) and purified by the RNeasy Mini Spin Columns (Qiagen). The integrity of RNA was checked with the Agilent Bioanalyser 2100 Eukaryote Total RNA Nano Series II system. The microarray experiment was performed using RNA isolated from the second leaf of 3-week-old seedlings and young-inflorescence at the booting stage. Pooled seedlings or young-inflorescence were used to construct each of three biological replicates for each plant line. The RNAs of the parental lines (*Ae. longissima* and *T. urartu*) were mixed at a ratio of 1:1 (quantity) to generate the empirical the *in vitro* “hybrids” counterpart of the synthetic allotetraploid line AT2. Microarray transcriptome profiling was performed by Affymetrix at the Gene Company, as described in the Gene Chip Expression Analysis Technical Manual. We noted that the recently published genome sequence of hexaploid wheat has annotated a total of 124,201 gene loci distributed nearly evenly across the homeologous chromosomes and subgenomes^57^. The Affymetrix GeneChip® Wheat Genome Array used in this study contains a total of 61,127 probes which are corresponded to 55,052 transcripts. To this end, we think that the number of genes represented by the Affymetrix GeneChip® Wheat Genome Array is representative. The microarray data have been submitted to the National Center for Biotechnology Information’s Omnibus repository (http://www.ncbi.nlm.nih.gov/geo/subs/) and are available under the accession number GSE65844.

### Microarray Data Normalization and Analysis

The raw CEL data were normalized with the robust multichip average method using the *R* software limma package. Genes that were differentially expressed among genotypes were identified by performing the t-moderated test ebayes^58^, and the raw *P* values were adjusted for multiple testing effects by the Benjamini and Yekutieli method (false discovery rate, or FDR < 0.05). The present or absent calls of each probe set were determined by the MAS5 method using *R* package. Differently expressed genes that did not show present calls in all three biological replications of at least one genotype were excluded from further analysis^59^. In total, 24,494 and 26,197 genes were detected as expressed in the leaf and young-inflorescence tissues, respectively.

### Subgenome-Specific cDNA Pyrosequencing

As the array used in this study was not designed to distinguish the subgenomes of polyploid wheat at either the tetraploid or hexaploid levels, we therefore selected >200 genes to analyze the homeolog expression changes by the cDNA pyrosequencing technique. The protocol was essentially as reported^60^ with modifications^54^. Primers of protein-coding genes for which diagnostic SNPs exist between the two subgenomes (S^l^ and A) were designed (Data Set S2) and verified by the pyrosequencing system (PyroMarkID Q96; Biotage). The two diploid progenitors, *Ae. longissima* and (S^l^S^l^) and *T. urartu* (AA), were used to assign SNPs to the S^l^ and A subgenomes, respectively. Biotin labeled PCR products were immobilized on streptavidin-coated paramagnetic beads. Capture of biotinylated single-stranded PCR products, annealing of the sequencing primer, and solid-phase pyrosequencing were performed following the manufacturer’s recommendations.

### Gene Annotation

Gene ontology (GO) annotations were performed using agriGO (http://bioinfo.cau.edu.cn/agriGO/index.php); the Singular Enrichment Analysis tool was used for GO annotations and significant GO term enrichment analysis^61^, which computed GO term enrichment in one set of genes by comparing it with another set, named the target and reference list, respectively. Enrichment was calculated by Fisher’s exact test with Hochberg’s multitest adjustment (FDR, *P* < 0.05).

### Phenotyping of Seedling Biomass and Seed-Setting

Greenhouse-grown plants of AT2 along with its diploid parents *Ae. longissima* and *T. urartu*, one natural wild tetraploid wheat, *T. turgidum ssp. dicoccoides* (TD) and one domesticated tetraploid wheat, *T. turgidum ssp.* *durum* (TTR13) were used to measure seedling biomass (3-week-old) and score seed-setting at maturity. Whole seedlings from all genotypes were harvested to measure plant height (the length between the plants above ground to apical meristem). The seedling biomass was determined after drying at 80 °C to constant mass. Seedlings of 12 plants in three biological replicates were weighed individually, and mean and SD were calculated. The rate of seed-setting was calculated by diving the number of filled seeds by the total number of kernels of all spikes of a given plant^54^.

### Accession Numbers

Detailed information including gene accession numbers of all genes studied in this article is listed (Data Sets 1 & 2). The microarray data have been submitted to the National Center for Biotechnology Information’s Omnibus repository (http://www.ncbi.nlm.nih.gov/geo/subs/) and are available under the accession number GSE65844.

## Additional Information

**How to cite this article**: Zhang, H. *et al.* Transcriptome shock invokes disruption of parental expression-conserved genes in tetraploid wheat. *Sci. Rep.* **6**, 26363; doi: 10.1038/srep26363 (2016).

## Supplementary Material

Supplementary Information

Supplementary Dataset 1

Supplementary Dataset 2
