# Supplementary material for: Transcriptome shock invokes disruption of parental expression-conserved genes in tetraploid wheat
Source: Sci Rep. 2016 May 20;6:26363. doi: 10.1038/srep26363 (PMC4873831; doi:10.1038/srep26363)
Supplement: Supplementary Information [file srep26363-s1.pdf]

Supplementary information for "Transcriptome shock invokes disruption of parental expression-conserved genes in tetraploid wheat"

Huakun Zhang[1,3*], Xiaowan Gou[1*], Ai Zhang[1], Xutong Wang[1], Na Zhao[1], Yuzhu Dong[1], Linfeng Li[1,2] & Bao Liu[1]

[1]Key Laboratory of Molecular Epigenetics of the Ministry of Education (MOE), Northeast Normal University, Changchun 130024, China

[2]Department of Biology, Washington University in St. Louis, MO 63130, USA

[3]Present address: John Innes Centre, Colney, Norwich NR4 7UH, United Kingdom

[*]These authors contributed equally to this study.

Correspondence and requests for materials should be addressed to L.F.L. (lilf241@nenu.edu.cn) or B.L. (baoliu@nenu.edu.cn)

Table of Contents

Supplemental Figure S1: Expression difference between the two diploid parents………S2

Supplemental Figure S2: Comparison of each of the additive and nonadditive expression pattern………………………………………………………………………………S3

Supplemental Table 1: Gene ontology analysis of over-TRE genes………………………S4

Supplemental Table 2: Inheritance and rewiring of subgenome homeolog expression in the leaf tissue of the synthetic allotetraploid wheat……………………………………………S5

Supplemental Table 3: Inheritance and rewiring of subgenome homeolog expression in the young inflorescence tissue of the synthetic allotetraploid wheat…………………………S6

Supplemental Table 4: The material list of the 18 wild and 14 domesticated nature tetraploid wheat ……………………………………………………………………………S7

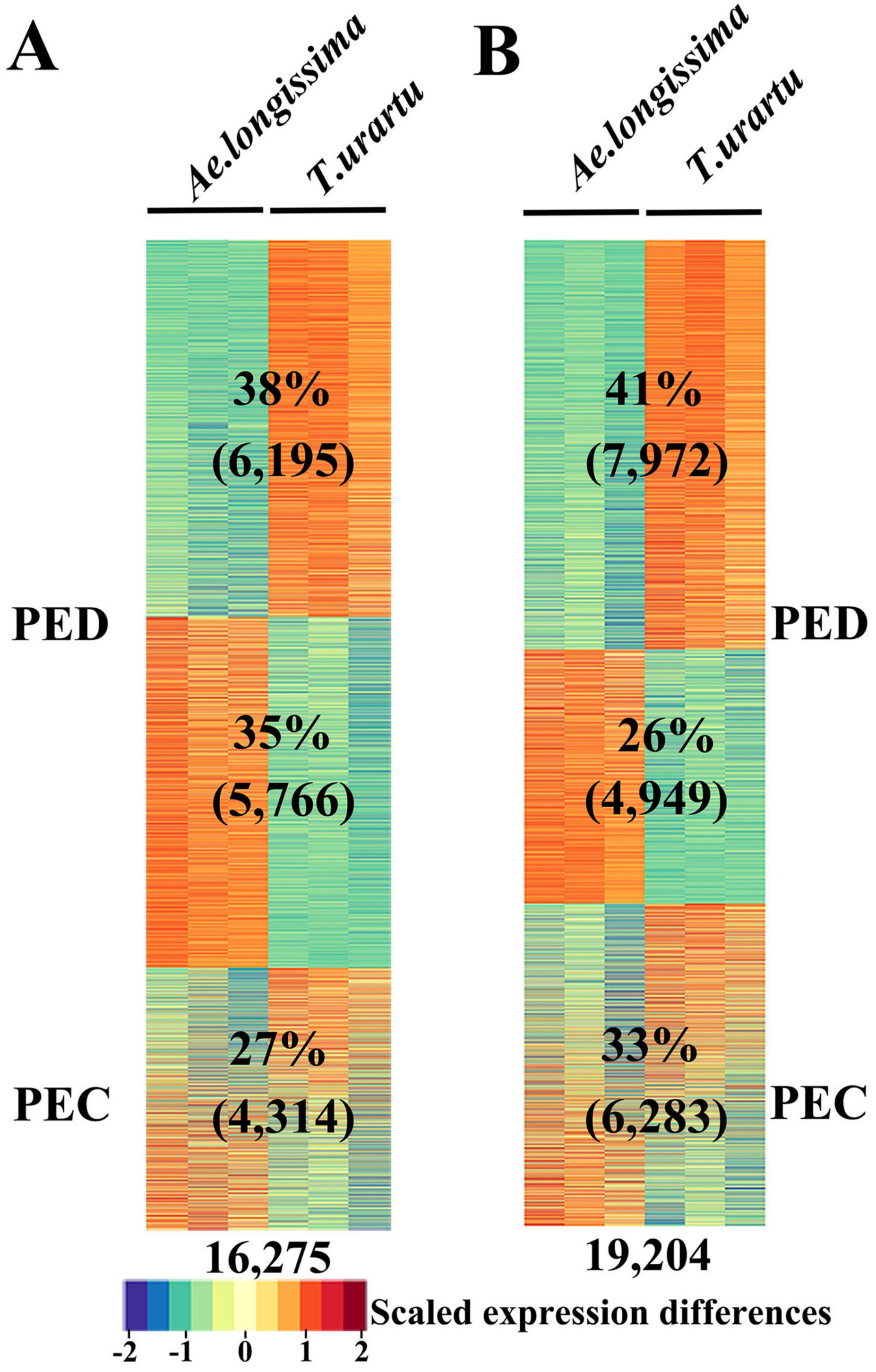

Supplemental Figure S1. Expression difference between the two diploid parental species in the leaf **(A)** and young inflorescence **(B)**, based on the microarray data. All three biological replicates of each parent are shown. The numbers and proportions of genes showing parental expression difference (PED) and parental expression conserved (PEC) patterns are given. Furthermore, the PED genes can be divided into two parts, the S^lS^l<AA and S^lS^l>AA. The numbers of the total expressing genes are shown at the bottom. The color key is indicated.

	Additive expression		Nonadditive expression							
			ELD				TRE			
			A-ELD		S^l-ELD		Over-TRE		Under-TRE	
		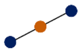	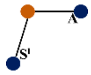	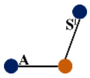	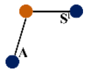	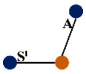	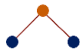	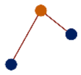	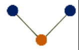	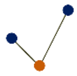
**L S_5-S_8**	3174	5095	1252	260	1136	192	1811	1447	624	346
	3899	5822	1222	223	1104	248	1748	1131	463	191
	3945	7090	765	317	520	239	821	501	585	226
	3441	4800	1033	338	1097	315	2249	1675	671	482
**YI S_5-S_8**	5284	7546	878	180	929	93	1161	685	346	385
	6036	8055	603	254	717	268	1131	631	190	115
	5982	8740	409	273	731	180	334	181	254	194
	6174	8229	542	255	645	262	1016	577	175	107

Supplemental Figure S2. Comparison of each of the additive and nonadditive expression patterns at each of the four selfed generations (S_5 to S_8) in leaf and young-inflorescence tissues of a synthetic allotetraploid wheat AT2. ELD: expression level dominance; TRE: transgressive expression; L: leaf; YI: young-inflorescence**.** The blue dot represents the diploid parents (*T. urartu*: AA genome, *Ae. longissima*: S^lS^l genome), the orange dot represents the synthesized allotetraploid.

Supplemental Table 1. Gene ontology (GO) analysis of over-TRE genes that showed transgenerational conserved nonadditive expression that are originated from parental expression-conserved genes (PEC) in a synthetic allotetraploid wheat AT2

Leaf

Ontology	GO Term	FDR
CC	endoplasmic reticulum	0.0012
MF	ATP binding	0.0047
BP	protein transport	0.017
BP	protein modification process	0.039

Young-inflorescence

Ontology	GO Term	FDR
MF	uroporphyrinogen decarboxylase activity	0.011

Shown are significantly enriched GO terms (Fisher test FDR < 0.05). CC: cellular component; MF: molecular function; BP: biological process.

Supplemental Table 2. Inheritance and rewiring of subgenome homeolog expression in each of the four selfed generations (S_5 to S_8) in leaf tissue of a synthetic allotetraploid wheat AT2

Leaf	Parents[a]	Progeny[b]	S_5	S_6	S_7	S_8
Parental condition	$S^l = A$	$S^l = A$	23 (15.1%)	27 (18.1%)	14 (9.2%)	27 (17.9%)
Parental condition	$S^l < A$	$S^l < A$	32 (21%)	29 (19.5%)	18 (11.9%)	26 (17.2%)
Parental condition	$S^l > A$	$S^l > A$	30 (19.7%)	12 (8%)	21 (13.8%)	14 (9.3%)
No bias in progeny	$S^l < A$	$S^l = A$	15 (9.9%)	18 (12%)	16 (10.5%)	16 (10.6%)
No bias in progeny	$S^l > A$	$S^l = A$	22 (14.5%)	46 (30.9%)	28 (18.4%)	44 (29.1%)
Novel bias in progeny	$S^l = A$	$S^l < A$	11 (7.2%)	8 (5.4%)	18 (11.9%)	8 (5.3%)
Novel bias in progeny	$S^l = A$	$S^l > A$	7 (4.7%)	5 (3.4%)	9 (5.9%)	5 (3.3%)
Novel bias in progeny	$S^l > A$	$S^l < A$	12 (7.9%)	4 (2.7%)	16 (10.5%)	7 (4.6%)
Novel bias in progeny	$S^l < A$	$S^l > A$	0 (0%)	0 (0%)	12 (7.9%)	4 (2.7%)
No bias	41 (26.8%)		60 (39.5%)	91 (61.1%)	58 (38.2%)	87 (57.6%)
Overall A-bias	47 (30.7%)		55 (36.2%)	41 (27.5%)	52 (34.2%)	41 (27.2%)
Overall S^l-bias	65 (42.5%)		37 (24.3%)	17 (11.4%)	42 (27.6%)	23 (15.2%)

$S^l = A$ denotes equal expression; $S^l < A$ and $S^l > A$ denotes subgenomes S^l-biased and A-biased expression, respectively.

[a] Comparison of the relative transcript contribution by subgenomes S^l and A in the *in vitro* "hybrids" (parental cDNA 1:1 mix) based on cDNA-pyrosequencing.

[b] Comparison of the relative transcript contribution by subgenomes S^l and A in the four successive generations of AT2 based on cDNA pyrosequencing.

Supplemental Table 3. Inheritance and rewiring of subgenome homeolog expression in each of the four selfed generations (S_5 to S_8) in young inflorescence tissue of a synthetic allotetraploid wheat AT2

Young inflorescence	Parents[a]	Progeny[b]	S_5	S_6	S_7	S_8
Parental condition	$S^l = A$	$S^l = A$	11 (7.5%)	13 (9.2%)	8 (5.6%)	12 (8.3%)
Parental condition	$S^l < A$	$S^l < A$	52 (35.4%)	43 (30.3%)	52 (36.1%)	42 (29.1%)
Parental condition	$S^l > A$	$S^l > A$	10 (6.8%)	9 (6.3%)	11 (7.6%)	8 (5.6%)
No bias in progeny	$S^l < A$	$S^l = A$	38 (25.8%)	51 (35.9%)	39 (27.1%)	53 (36.8%)
No bias in progeny	$S^l > A$	$S^l = A$	5 (3.4%)	5 (3.5%)	4 (2.8%)	6 (4.2%)
Novel bias in progeny	$S^l = A$	$S^l < A$	1 (0.7%)	1 (0.7%)	2 (1.4%)	1 (0.7%)
Novel bias in progeny	$S^l = A$	$S^l > A$	12 (8.2%)	8 (5.6%)	12 (8.3%)	9 (6.3%)
Novel bias in progeny	$S^l > A$	$S^l < A$	0 (0%)	0 (0%)	0 (0%)	0 (0%)
Novel bias in progeny	$S^l < A$	$S^l > A$	18 (12.2%)	12 (8.5%)	16 (11.1%)	13 (9%)
No bias	22 (15%)		54 (36.7%)	69 (48.6%)	51 (35.4%)	71 (49.5%)
Overall A-bias	109 (74.7%)		53 (37.3%)	44 (31%)	54 (37.5%)	43 (29.9%)
Overall S^l-bias	15 (10.3%)		40 (28.2%)	29 (20.4%)	39 (27.1%)	30 (20.8%)

$S^l = A$ denotes equal expression; $S^l < A$ and $S^l > A$ denotes subgenomes S^l-biased and A-biased expression, respectively.

[a] Comparison of the relative transcript contribution by subgenomes S^l and A in the *in vitro* "hybrids" (parental cDNA 1:1 mix) based on cDNA-pyrosequencing.

[b] Comparison of the relative transcript contribution by subgenomes S^l and A in the four successive generations of AT2 based on cDNA pyrosequencing.

Supplemental Table 4. A set of 18 wild (*dicoccoides*) and 14 domesticated (*durum*) nature tetraploid wheats, *T. turgidum*, derived from diverse origins

Dicoccoides		***Durum***	
**Designation**	**Origin**	**Designation**	**Origin**
TD	Israel	TTR13	Israel
W1	Mt. Hermon, Israel	Z1	Russia
W2	Rosh Pinna, Israel	Z2	Germany
W3	Tabigha, Israel	Z3	USA
W4	Bat-Shelomo, Israel	Z4	Israel
W5	Mt. Gilboa, Israel	Z5	Eritrea
W6	Mt. Gerizim, Israel	Z6	Italy
W7	Amirim, Israel	Z7	France
W8	Kokhav hashahar, Israel	Z8	Canada
W9	Bet-Oren, Israel	Z9	Ukraine
W10	Givat-Koach, Israel	Z10	Kazakhstan
W11	Gitit, Israel	Z11	Russia
W12	J'aba, Israel	Z12	Ethiopia
W13	Gamlla, Israel	Z13	Tunisia
W14	Tabigha, Israel		
W15	Tabigha, Israel		
W16	Yehudiyya, Israel		
W17	Diyarbakir, Turkey		
